# Efficiency of Hydroxycinnamic Phenolic Acids to Inhibit the Production of Ochratoxin A by *Aspergillus westerdijkiae* and *Penicillium verrucosum*

**DOI:** 10.3390/ijms21228548

**Published:** 2020-11-13

**Authors:** Saranyaphat Boonmee, Vessela Atanasova, Sylvain Chéreau, Gisèle Marchegay, Kevin D. Hyde, Florence Richard-Forget

**Affiliations:** 1 Center of Excellence in Fungal Research, Mae Fah Luang University, Chiang Rai 57100, Thailand; saranyaphat.khag2@gmail.com (S.B.); kdhyde3@gmail.com (K.D.H.); 2 School of Science, Mae Fah Luang University, Chiang Rai 57100, Thailand; 3 UR1264 Mycology and Food Safety Research Unit (MycSA), INRAE Research Centre, F-22882 Villenave d’Ornon, France; sylvain.chereau@inrae.fr (S.C.); gisele.marchegay@inra.fr (G.M.); florence.forget@inrae.fr (F.R.-F.)

**Keywords:** Aspergillus westerdijkiae, Penicillium verrucosum, ochratoxin A, phenolic acids

## Abstract

Ochratoxin A (OTA) is one of the worldwide most important mycotoxins in terms of health and agroeconomic consequences. With the aim to promote the use of phytochemicals as alternatives to synthetic fungicides, the effect of hydroxycinnamic acids on the fungal growth and OTA yield by two major OTA-producing species was investigated. After a first step dedicated to the definition of most suitable culture conditions, the impact of 0.5 mM ferulic (FER), *p*-coumaric (COUM), caffeic and chlorogenic acids was evaluated on *Aspergillus westerdijkiae* and *Penicillium verrucosum*. Whereas no fungal growth reduction was observed regardless of the phenolic acid and fungal isolate, our results demonstrated the capacity of FER and COUM to inhibit OTA production. The most efficient compound was FER that led to a 70% reduction of OTA yielded by *P. verrucosum* and, although not statistically significant, a 35% inhibition of OTA produced by *A. westerdijkiae*. To further investigate the bioactivity of FER and COUM, their metabolic fate was characterized in fungal broths. The capacity of *P. verrucosum* to metabolize FER and COUM through a C_2_-clivage type degradation was demonstrated. Overall, our data support the potential use of FER to prevent OTA contamination and reduce the use of synthetic pesticides.

## 1. Introduction

Contamination of food and feeds with mycotoxins is a global issue. According to a commonly cited estimate ascribed to Food and Agriculture Organization (FAO), 25% of the world′s crops could be contaminated with mycotoxins above the regulatory limits, leading to annual losses close to 1 billion metric tons [1]. This prevalence was recently confirmed by Eskola et al. [2] who also reported that 60–80% of agricultural commodities contain mycotoxins above detectable levels. With aflatoxins, patulin, trichothecenes, zearalenone, and fumonisins, ochratoxin A (OTA) is one of the worldwide most important mycotoxins in terms of effects on health and agroeconomic consequences. OTA is synthetized by several species of the *Aspergillus* and *Penicillium* fungal genera. The most common OTA-producing *Aspergillus* species are *Aspergillus ochraceus*, *Aspergillus westerdijkiae, Aspergillus niger,* and *Aspergillus carbonarius* while the well ascertained OTA-producers within the *Penicillium* genus are *Penicillium verrucosum* and *Penicillium nordicum* [3]. In tropical and subtropical areas characterized by elevated temperatures and humidity rates, contamination of food and feedstuffs is mainly due to *Aspergillus* species whereas in temperate climates, *P. verrucosum* is the major species responsible for the presence of OTA. OTA can be produced by *Aspergillus* during the storage of a broad range of foods such as cereals, coffee, cocoa, spices, dried fruits, and of animal feeds when environmental conditions in storage facilities (temperature, humidity) are not efficiently controlled. Besides, OTA can be produced by *A. carbonarius* and *A. niger* in grapes during the cultivation of vines, the two former species being frequently isolated from the same batches of berries [4]. *P. verrucosum* that is capable of growth at low temperature (optimum 20 °C) is the major species responsible for the occurrence of OTA in European cereals and cereal-based products. Actually, the consumption of cereal-derived products represents the main source of exposure to OTA for European citizens [5].

OTA is classified by the International Agency for Research on Cancer (IARC) in group 2B, i.e., as a possible human carcinogen. In addition, this toxin has shown to exhibit nephrotoxic, teratogenic, and immunosuppressive properties [6,7]. Notably, OTA has been supposedly implicated as a causal agent of the Balkan Endemic Nephropathy, a chronic tubulointerstitial disease associated with urothelial cancer affecting humans [8]. The knowledge that OTA can have serious deleterious effects on humans and animals has led many countries to regulate its occurrence in food and feed. In Europe, the 1881/2006 regulation sets maximal levels of OTA in various foodstuffs.

OTA is relatively stable once accumulated in raw agricultural materials and its complete elimination from commodities is not realistically achievable with the industrial processes currently available. Prevention of fungal growth and OTA production during plant cultivation or storage should be the preferred strategy to impede its harmful effects on animal and human health. Combined with good agricultural practices and proper management during harvest and storage, the use of fungicides is a key factor in the integrated management strategies aiming at controlling the OTA mycotoxin. The repeated use of chemical fungicides over decades has however disrupted natural biological systems and resulted in the development of fungal resistance. Moreover, fungicides have acknowledged undesirable effects on nontarget organisms and fostered environmental and human health concerns. In an attempt to reduce the use of synthetic fungicides and reply to European directives such as the 2009/128/European Commission (EC), there is an urgent need to develop innovative natural product-based strategies to durably control the infection of harvests by toxigenic fungi and their contamination with mycotoxins. In recent years, accelerated efforts have been undertaken in the screening of plant derived substances with a greatest attention to plant secondary metabolites involved in the plant chemical defensive response deployed to counteract fungal infection. Notably, some phenolic acids have been reported as potential biochemical resistance factors in several pathosystems, such as apple—*Penicillium expansum* [9]; bean—*Aspergillus* species producing aflatoxin [10]; maize—*Fusarium verticillioides* [11]; tomato—*Alternaria alternata* [12]. In addition to their contribution to the reinforcement of plant structural components acting as a mechanical barrier and their role as signaling molecules, phenolic acids can participate in plant defense mechanisms through direct interference with toxigenic fungi [13]. There is notably a large body of evidence that supports the inhibitory activities of hydroxycinnamic acid derivatives, mainly *p*-coumaric (COUM), ferulic (FER), caffeic (CAF), and chlorogenic (CHLO) acids, towards the growth of toxigenic fungi and the biosynthesis of mycotoxins including deoxynivalenol, type A trichothecenes, fumonisins, and aflatoxin [14,15]. The effects of phenolic acids on OTA-producing species are however much less documented and, in most cases, limited to their fungicide activity against *Aspergillus* species [16,17]. A small number of publications focusing exclusively on OTA-producing *Aspergillus* isolates has corroborated the potential of CAF, FER, and COUM acids to inhibit the production of OTA [14,18,19]. To our knowledge, no data as regards the way phenolic acids could modulate the production of OTA by *Penicillium* species have been published so far.

The present work was aimed at providing new insights on the capacity of hydroxycinnamic acids (CHLO, CAF, FER, and COUM) to interfere with the production of OTA by two of the most relevant OTA-producing species in Europe, *A*. *westerdijkiae* and *P. verrucosum*. To a better understanding of the mechanisms involved in the modulation of OTA yields, the capacity of toxigenic fungi to biotransform hydroxycinnamic acids was investigated. A first step was implemented to select the most suitable fungal culture conditions for investigating the effects of phenolics acids. To this end, three criteria were addressed: (i) a significant fungal growth, (ii) a substantial OTA yield, (iii) a pH value lower or close to the phenolic acid pKa values throughout the incubation period. Actually, bioactivity of phenolic acids is assumed to be primarily linked to the concentration of nondissociated carboxyl groups [20,21].

## 2. Results

### 2.1. Search for Suitable Culture Conditions to Investigate the Effects of Hydroxycinnamic Acids on Fungal Growth and OTA Production by A. westerdijkiae and P. verrucosum

Five media previously reported as conducive to OTA production [22,23], tested in both static and agitated conditions, were considered. Fungal biomass and OTA accumulation were quantified in each culture condition. pH was measured at day 0 and at the end of the fungal culture time, i.e., 11 days after inoculation.

#### 2.1.1. Comparison of Fungal Biomass in the Five Tested Media under Static and Agitated Conditions

Data regarding the amounts of fungal biomass of *A. westerdijkiae* and *P. verrucosum* accumulated after 11 days of incubation in the five media under static and agitated cultures are gathered in Table 1. A significant growth (values >100 mg) was observed for both species regardless of the medium used. The lowest biomass yield (values <200 mg) was obtained in static Czapek Yeast Autolysate medium (CYA) for both *A. westerdijkiae* and *P. verrucosum* strains whereas the maximal biomass was yielded in agitated Yeast Extract Sucrose_A (YES_A) and YES_B media with 1484.33 and 1057.87 mg for *A. westerdijkiae*, and 1135.37 and 880.77 mg for *P. verrucosum*, respectively (Table 1). As expected, the maximum growth for both species was recorded in agitated conditions. Interestingly, static conditions were shown to lead to lower standard deviation values compared to the agitated ones.

#### 2.1.2. OTA Production by *A. westerdijkiae* and *P. verrucosum* in the Five Tested Media under Static and Agitated Conditions

**Figure 1:**
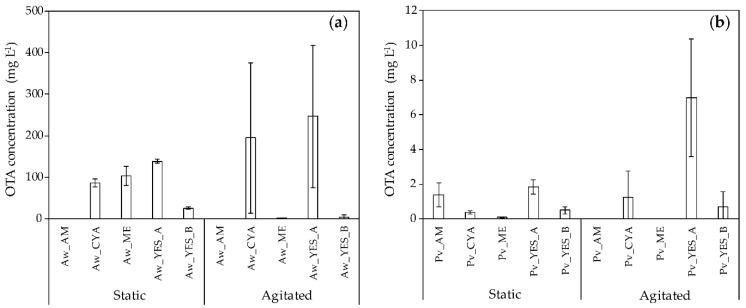
Ochratoxin A (OTA) production in Adye and Mateles (AM), Czapek Yeast Autolysate (CYA), Malt Extract (ME), Yeast Extract Sucrose_A (YES_A), and YES_B media by (**a**) *Aspergillus westerdijkiae* (Aw) and (**b**) *Penicillium verrucosum* (Pv) under static and agitated conditions. Data are means ± standard deviation using three biological replicates.

Concentrations of OTA produced by *A. westerdijkiae* and *P. verrucosum* after 11 days of incubation in the different culture conditions are reported in Figure 1a,b.

For each medium and for both static and agitated conditions, *A. westerdijkiae* produced far more OTA than *P. verrucosum* (Figure 1a,b). The highest amounts of OTA yielded by *P. verrucosum* were 1.84 mg L^−1^ (*P. verrucosum*_ YES_A broths, Pv_YES_A) in static conditions and 6.99 mg L^−1^ (Pv_YES_A) in agitated ones, whereas, for *A. westerdijkiae*, these values reached 138.54 mg L^−1^ (*A. westerdijkiae*_ YES_A broths, Aw_YES_A) in static conditions and 246.72 mg L^−1^ (Aw_YES_A) in agitated ones. The effects of the medium composition on OTA yields in agitated conditions were shown to follow similar patterns for both species, i.e., highest amounts in CYA and YES_A and no OTA or low OTA concentrations in Adye and Mateles medium (AM), Malt Extract medium (ME), and YES_B. In static conditions, all the used media, with the exception of AM inoculated by *A. westerdijkiae,* were shown to allow OTA accumulation in quantifiable amounts. As previously observed for fungal biomass, standard deviation values for OTA levels produced by both species, *A. westerdijkiae* and *P. verrucosum*, were significantly lower in static conditions compared to those measured in agitated conditions (Figure 1a,b).

#### 2.1.3. Evolution of pH in the Inoculated Media

There is a large body of evidence suggesting that antimicrobial activity of phenolic acids is linked to the concentration of their respective nondissociated carboxyl group and is therefore favored at low pH, below or close to their pKa values [20,21]. pKa values of the phenolic acids considered in the present study are 4.58 for FER, 4.0 for COUM, 4.62 for CAF, and 3.33 for CHLO. The pH values measured in each of the culture conditions, non-inoculated or inoculated by *A. westerdijkiae* and *P. verrucosum*, at day 0 and day 11 are reported in Table 1.

Initial pH was acidic in AM and ME media and close to neutral in CYA, YES_A, and YES_B media with values of 7.2, 6.6, and 6.7, respectively. No change in pH values was observed in non-inoculated control media after 11 days of incubation at 25 °C. For most of the modalities, the fungal growth was accompanied by a slight increase or decrease in pH values, ranging between 0.2 and 1.3 unit. Substantial differences between pH values at day 0 and day 11 were, however, observed in few modalities: static AM inoculated by either *A. westerdijkiae* or *P. verrucosum* (pH decrease of 3.1 and 2.1 units, respectively) and agitated YES_B inoculated by *A. westerdijkiae* (pH decrease of 2.2 units).

Altogether, the previous data led us to select the ME medium and static conditions for continuation of the study. Indeed, this modality was the only one that meets three major criteria required to investigate the effect of phenolic acids: (i) a substantial growth and quantifiable amounts of OTA for the two model species (103.80 mg L^−1^ for *A. westerdijkiae* and 0.08 mg L^−1^ for *P. verrucosum*), (ii) reduced standard deviation values between replicates, and (iii) pH values close to the pKas of the studied phenolic acids throughout the incubation period.

### 2.2. In Vitro Effect of Hydroxycinnamic Acids on Fungal Growth and OTA Production by A. westerdijkiae and P. verrucosum

The effect of 0.5 mM FER, COUM, CAF, and CHLO on fungal growth and OTA biosynthesis by *A. westerdijkiae* and *P. verrucosum* was evaluated after 11 days of culture. The 0.5 mM concentration was chosen according to literature data regarding previously published anti-mycotoxin efficiencies of phenolic acids and their physiological levels in plant tissues [14]. Concentrations closed to 0.5 mM were reported for free CHLO in maize kernels at the earliest stages of grain development [24] or in coffee [25] and for free FER in wheat grains at maturity [26].

**Figure 2:**
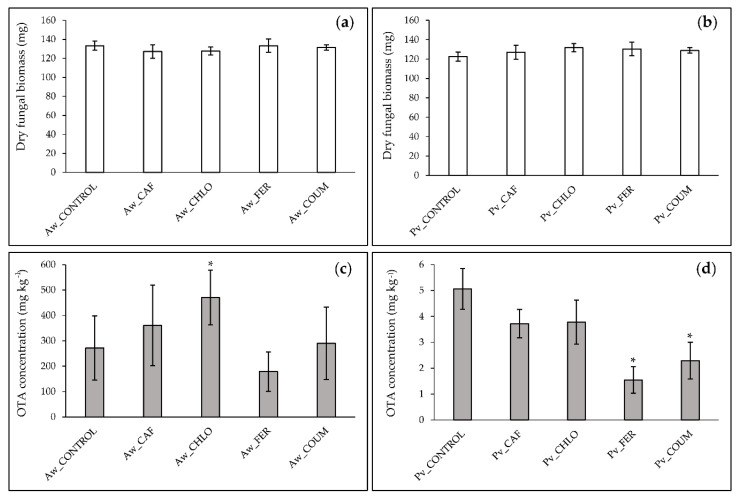
Effect of 0.5 mM caffeic (CAF), chlorogenic (CHLO), ferulic (FER), and *p*-coumaric (COUM) acids on fungal growth of (**a**) *Aspergillus westerdijkiae* (Aw) and (**b**) *Penicillium verrucosum* (Pv) and on ochratoxin A (OTA) production by (**c**) *A. westerdijkiae* and (**d**) *P. verrucosum*. Values are expressed as means ± standard deviation using four biological replicates. Asterix (*) indicates significant differences when compared with control (Student’s *t* test, control versus treated, *p* < 0.05).

Initial pH of the culture media (pH = 4.2) was not affected by phenolic acid treatments and final pH conditions did not vary between treatment, pH = 5.5 and 5.0 for *A. westerdijkiae* and *P. verrucosum* cultures, respectively. Figure 2a,b shows the impact of FER, COUM, CAF, and CHLO at 0.5 mM on fungal growth of *A. westerdijkiae* and *P. verrucosum*. The results revealed that the four phenolic acids at 0.5 mM did not affect the fungal growth, regardless of the fungal isolate.

The effect of the four studied phenolic acids on OTA production by *A. westerdijkiae* and *P. verrucosum* is reported in Figure 2c,d. Toxin yields in control cultures of *A. westerdijkiae* and *P. verrucosum* reached 271 and 5 mg kg^−1^ of dried mycelia, respectively. An increase in the amounts of OTA produced by *A. westerdijkiae* was observed in CAF and CHLO-treated cultures; this increase was however statistically significant only for the CHLO treatment. Our data indicated a reduction of OTA accumulation induced by FER in *A. westerdijkiae* and *P. verrucosum* broths; this reduction was only significant for *P. verrucosum* (inhibition rate close to 70%). A two-fold significant inhibition of OTA biosynthesis by *P. verrucosum* was also observed in COUM-supplemented cultures.

The former results suggest that FER and, with a lower efficiency, COUM could be promising inhibitors of OTA biosynthesis by *P. verrucosum*. We further explored the mechanisms involved in OTA inhibition by FER and COUM by investigating the effect of exogeneous phenolic acid throughout the fungal growth kinetic and studying their potential metabolization by *P. verrucosum.*

### 2.3. Kinetic Study of Fungal Growth, OTA Production, and Fate of Phenolic Acids in P. verrucosum Broths Supplemented with FER and COUM

**Figure 3:**
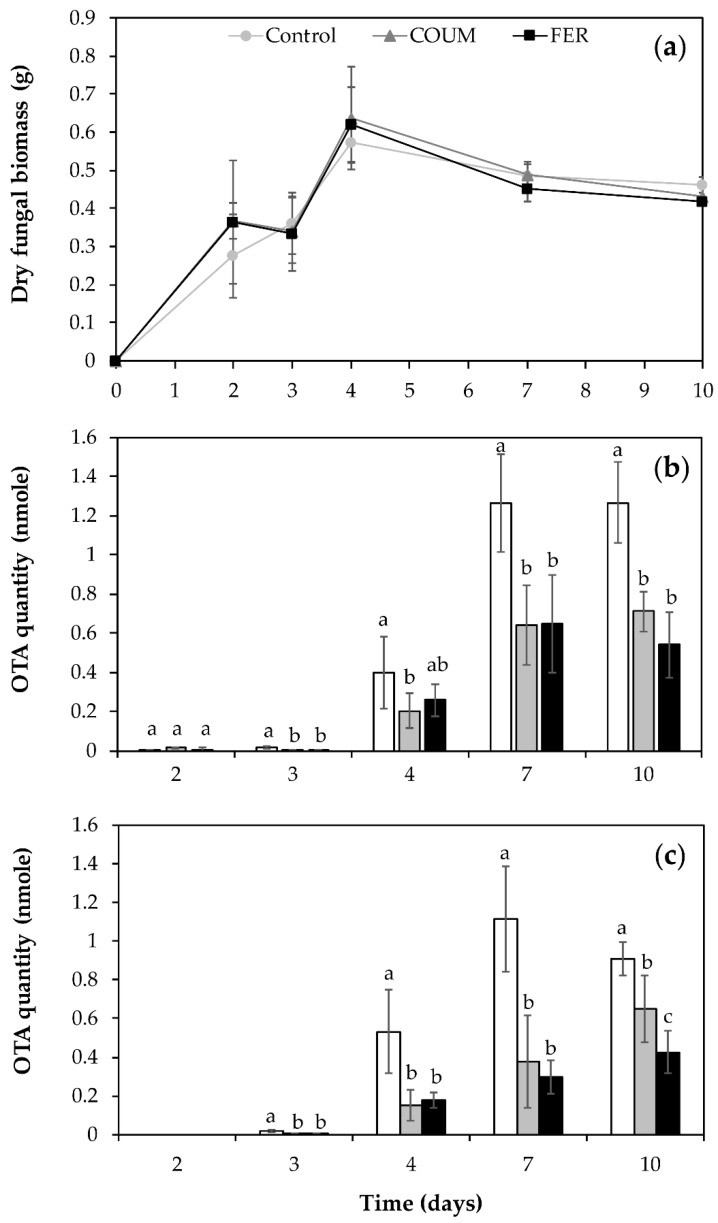
Kinetics of (**a**) fungal biomass and ochratoxin A (OTA) accumulated in (**b**) mycelia and in (**c**) supernatants of *Penicillium verrucosum* broths supplemented with 0.5 mM ferulic acid (FER) (black) and *p*-coumaric acid (COUM) (gray) or in non-supplemented control (white). Differences between control and tested phenolic acids in terms of OTA were determined separately for each day with multiple comparisons tests using the Dunn–Sidak method. Different letters indicate significant differences (*p* < 0.05). Values are expressed as means ± standard deviation using five biological replicates.

Fungal growth, OTA accumulation, and phenolic acid metabolic fate were monitored over 10 days after *P. verrucosum* inoculation in 0.5 mM FER and COUM-supplemented and non-supplemented cultures. To investigate whether the phenolic acid treatment could affect the secretion of OTA, OTA was assessed in both mycelia and culture supernatants. Results are gathered in Figure 3. Figure 3a describes the kinetic of biomass accumulation in control and in FER and COUM-treated cultures. Analysis of variance (ANOVA) indicated that the fungal growth was not significantly affected regardless of the treatment (*p* = 0.830). Biomass increased sharply during the first days of culture and reached maximum (close to 0.6 g) at day 4 to then remained stable.

Regarding OTA kinetics in mycelia (Figure 3b) and in supernatants (Figure 3c), the ANOVA tests evidenced significant differences among the treatments. The levels of OTA in supernatants and mycelia were very close with slightly higher values in mycelia.

In mycelia, OTA was first quantified at day 2 and its quantity increased regularly until day 7 to reach a maximum of 1.26, 0.65, and 0.64 nmole in control and treated conditions with FER and COUM, respectively. Between day 7 and day 10, OTA levels were constant in control mycelia and in the presence of COUM and FER. After 10 days of incubation, OTA in mycelia was reduced by 57% and 44% in the presence of FER and COUM, respectively. Overall, the percentages of OTA inhibition induced by FER and COUM supplementation decreased with time.

Regarding culture supernatants, OTA was first quantified at day 3 after inoculation. The kinetic of OTA accumulation in control cultures was nearly linear between day 3 and day 7 to reach a maximum of 1.11 nmole. Between day 7 and day 10, OTA decreased slightly. In FER and COUM-supplemented broths, OTA accumulation seemed to evolve differently from what was observed in control conditions. Actually, the maximal amounts were reached at the end of incubation. After 10 days of incubation, OTA levels in supernatants were reduced by 53% and 29% in the presence of FER and COUM, respectively. As observed for the mycelia, the inhibition percentages induced by exogeneous FER and COUM decreased with time: 86% at day 3, stable between day 4 and day 7 (67% and 73%) and 53% at day 10 for FER and 79% at day 3, 72% at day 4, 66% at day 7, and 29% at day 10 for COUM.

According to the previous results, the inhibition of OTA production assessed in mycelia and supernatants showed similar patterns, which does not support the hypothesis of an inhibition of OTA secretion by exogeneous phenolic acid.

**Figure 4:**
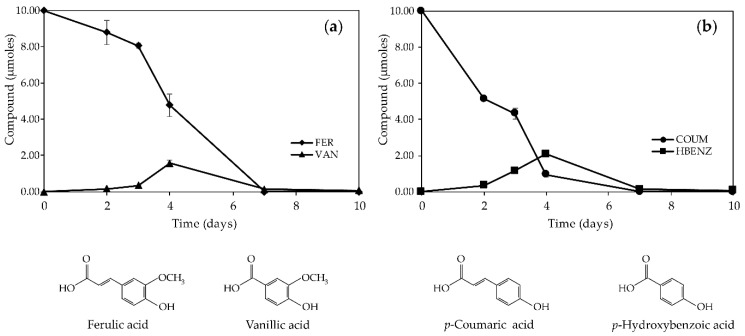
Kinetics and chemical structure of (**a**) ferulic acid (FER), (**b**) *p*-coumaric acid (COUM), and their metabolites in the supernatants of *Penicillium verrucosum* cultures supplemented with 0.5 mM of FER and COUM. Values are expressed as means ± standard deviation using five biological replicates. VAN—vanillic acid; HBENZ—*p*-hydroxybenzoic acid.

The metabolization pathways of FER and COUM were investigated in *P. verrucosum* broths. Results are gathered in Figure 4a,b. In supernatants of non-inoculated cultures, levels of FER and COUM were stable over the culture time. In FER-supplemented and inoculated supernatants, a gradual decrease in FER (8.79 µmoles at day 2, 8.06 µmoles at day 3, and 4.78 µmoles at day 4) during the first days of cultures was evidenced, resulting in unquantifiable levels of FER at day 7 (Figure 4a).

Liquid chromatography/diode-array detector (LC/DAD) profiles of the supernatants monitored at 260 nm showed the presence of one additional peak that was identified as vanillic acid (VAN) on the basis of its retention time and UV spectrum of a reference standard. VAN was first quantified at day 2, reached a maximum of 1.56 µmole at day 4, and decreased to 0.07 µmole at the end of the culture. VAN was not detected in non-inoculated supernatants supplemented with FER, suggesting that its production could be related to FER metabolization by *P. verrucosum*. The unbalanced ratio between FER disappearance (9.06 µmoles at day 4) and VAN accumulation (1.56 µmole at day 4) suggests the occurrence of additional mechanisms underpinning the decrease in FER.

Regarding COUM-supplemented and *P. verrucosum*-inoculated broths, a drastic decrease in the COUM quantity (close to 90%) was observed during the first 4 days of culture to reach unquantifiable values at day 7 (Figure 4b). On LC/DAD profiles recorded at 260 nm, one major peak was observed. This peak was ascribed to *p*-hydroxybenzoic acid (HBENZ) in comparison with retention time and UV spectrum of a commercial standard. HBENZ was shown to accumulate between day 0 and day 4, and then to decrease to reach 0.07 µmole at day 10. Since HBENZ was not detected in non-inoculated supernatants supplemented with COUM, our results suggest that *P. verrucosum* is the cause of COUM biotransformation in HBENZ. As previously concluded for FER decrease, the amount of accumulated HBENZ could not explain the 90% of COUM disappearance indicating the involvement of additional mechanisms.

## 3. Discussion

Phenolic acids are a subclass of phenolic compounds with reported antifungal and mycotoxin inhibitory activities against several mycotoxin-producing pathogens including *Fusarium graminearum*/deoxynivalenol [15], *Fusarium verticillioides*/fumonisins [11], *Aspergillus flavus*/aflatoxins [17], and hold promise for application as biofungicides or as preservatives during the storage of raw agricultural commodities. However, with the exception of few studies focusing on *A. carbonarius* and *A. ochraceus* [19,27], the capacity of phenolic acids to modulate the production of OTA has been little explored. In particular, the way phenolic acids could interfere with the production of OTA by *P. verrucosum* and *A. westerdijkiae* that are key species responsible for OTA contamination of European food and feedstuffs is poorly documented.

The present study was designed to address this knowledge gap. Through the implementation of a series of in vitro experiments, the effect of most abundant hydroxycinnamic phenolic acids occurring in various agricultural products such as cereals, maize, coffee, spices, or grape on OTA production by *A. westerdijkiae* and *P. verrucosum* was investigated. For the first time, our results demonstrated a clear inhibitory effect of FER and COUM on OTA biosynthesis by *P. verrucosum*. Inhibition rates close to 70% and 50% were obtained with 0.5 mM FER and COUM, respectively. Our data also supported the potential of FER to reduce the production of OTA by *A. westerdijikae.* Although not statistically significant, a 35% decrease in OTA yielded by *A. westerdijkiae* was observed in FER-supplemented media. Conversely to FER and COUM, no inhibition effect was evidenced when CAF or CHLO were supplemented to the broths. Altogether, the obtained data were in accordance with the few existing results that have proved the capacity of phenolic compounds to reduce OTA production by *A. carbonarius* and *A. ochraceus*, including total phenolic extracts from various plants and herbs [28], flavonoid compounds [29], catechin, and different phenolic acids [27].

To date, regardless of the phenolic compound and the mycotoxin biosynthesis pathway, the exact mechanisms by which mycotoxin production can be inhibited is not fully elucidated [14,30]. One of the most commonly raised hypotheses to explain the inhibitory effect of FER and COUM is related to the irreversible changes these molecules can cause to microbial membranes through hydrophobicity modifications, changes in negative surface charge, and formation of pores leading to consequent leakage of essential intracellular constituents [31]. The membrane-disrupting activity of hydroxycinnamic acids is linked to their lipophilicity and is increased for pH values lower or closed to their pKa, i.e., when the nondissociated forms of phenolic acids are predominant [20,21]. The pKa values of FER and COUM are 4.58 and 4.64, respectively. pH value of the ME medium was 4.2 before inoculation and reached 5 and 5.5 in *P. verrucosum* and *A. westerdijkiae* 11-day old cultures, respectively. This difference of 0.5 unit could be one rationale explaining the weaker bioactivity of FER and COUM observed in *A. westerdijkiae* broths.

A second frequently raised hypothesis is related to the antioxidant properties of phenolic compounds that are assumed to be primary factors for their antimycotoxin activity. Indeed, there is converging evidence indicating that the redox balance is one of the most crucial factors for the regulation of mycotoxin biosynthesis and that exposure to reactive oxygen species increases the production of mycotoxins by fungi [32,33]. Response to oxidative stress and OTA production by *Aspergillus* species have also been proved to be intertwined [34]. On a mechanistic side, the Aoyap1 transcription factor, a homolog of Yap-1 from yeast, has been evidenced to regulate OTA biosynthesis by controlling cell redox balance in *A. ochraceus* [35]. Treatment by oxidative stressors such as menadione [36] or carbon tetrachloride [35] have been reported to trigger the synthesis of OTA in *A. carbonarius* and *A. ochraceus*. Similarly, by-products of lipid oxidation catalyzed by lipoxygenases have been shown to enhance the yield of OTA by *A. ochraceous* [37]. Hydroxycinnamic acids that are acknowledged as potent antioxidant compounds due to their capacity to quench oxygen free radicals but also to interfere with various oxidative enzymatic activities including lipoxygenases [38], may reduce or suppress the oxidative stress upstream signal that governs the production of OTA. However, while the effect observed with FER and COUM acids supports the link between antioxidative properties and “anti-ochratoxin” activity, the lack of OTA modulation with CAF, one of the most potent antioxidants among hydroxycinnamic acids [38], demonstrates that antioxidant potential is not sufficient to explain the regulation of OTA biosynthesis. Moreover, while the biosynthesis of OTA by *Aspergillus* species has been shown to be increased by oxidative stress, an opposite effect has been reported regarding OTA yielded by *P. verrucosum* [39]. Actually, the previous publication indicated that application of an oxidative stress led to a reduction of the production of OTA by *P. verrucosum*, whereas the synthesis of citrinin (another mycotoxin produced by this fungal species) was enhanced. According to Schmidt-Heydt et al. [39], the biosynthesis of OTA and citrinin by *P. verrucosum* are interlinked and the induction of citrinin yield occurs at the expense of OTA. The former results are not consistent with the assumption that antioxidant properties of FER and COUM could be responsible for their capacity to inhibit the production of OTA by *P. verrucosum*. Besides, the different effects of oxidative stress on OTA yield according to the producing species could partially explain the differences in FER and COUM efficiency to reduce OTA production between *P. verrucosum* and *A. westerdijkiae* cultures.

Lastly but not least, phenolic compounds can inhibit the biosynthesis of mycotoxins by downregulating the expression of key biosynthetic genes as suggested by several reports [40,41]. Regarding OTA production, such a mechanism has been proposed to explain the bioactivity of cinnamaldehyde, a natural plant substance derived from cinnamon: a significant reduction of the expression of OTA biosynthetic and regulatory genes in *A. ochraceus* was induced by cinnamaldehyde exposure [42].

In order to support the application of FER and COUM acids as a preservative solution for OTA control during the storage of grains and anticipate fungal resistance, the capacity of *P. verrucosum* to degrade and detoxify FER and COUM acids was investigated. Very few information as regards the capacity of *Penicillium* species to metabolize phenolic acids is available. Indeed, a vast majority of the published studies addressing the biotransformation of phenolic acids by filamentous fungi comes from studies targeting *Aspergillus* strains [43,44]. Our work reports for the first time the ability of *P. verrucosum* to biotransform FER and COUM. These findings were supported by the detection and quantification of degradation metabolites: VAN in FER-supplemented cultures and HBENZ in COUM-supplemented cultures (Figure 4). Degradation of FER and COUM were shown to occur most actively during the 5 first days of culture, i.e., during the exponential phase of mycelial growth (Figure 3 and Figure 4). Bioconversion of FER into VAN and of COUM into HBENZ by *P. verrucosum* are consistent with a C_2_-clivage type degradation. Due to the wide range of industrial and food applications and mainly the production of vanillin, one of the most important flavor molecules, the microbial degradation of FER has been the subject of intense research and different pathways for its bioconversion into VAN have been proposed. In the proposed metabolic routes reported for *Pycnoporus cinnabarinus* [45], *Aspergillus niger*, and *Phanerochaete crysosporum* [44], the propenoic side chain of FER is oxidatively cleaved to yield VAN, that can be further oxidized into vanillin or decarboxylated into methoxyquinone. In *Schizophyllum commune*, the proposed bioconversion process starts by the decarboxylation of FER into 4-vinylguaicol followed by its conversion into vanillin and finally the reduction of vanillin into VAN [46]. In our experiments, while more than 5 µmoles of FER have disappeared from culture broths after 4 days of incubation, only 1.8 µmole of VAN was recovered. This unbalanced ratio can suggest the further biotransformation of VAN into metabolites even though no additional metabolites were evidenced with the chromatographic method we used. Actually, neither vanillin nor methoxyquinone were detected in the FER-supplemented broths. As regards the biotransformation of COUM by *P. verrucosum*, the detection of HBENZ in inoculated cultures suggests a catabolic route similar to that previously reported for *Paecilomyces variotii* [47]. As for FER, the amount of quantified HBENZ was not sufficient to explain the whole decrease in COUM, raising the possibility of a further biotransformation of HBENZ.

The present study has demonstrated the clear inhibitory effect of FER on OTA production by *P. verrucosum* and has underlined its potential to be used in storage strategies to reduce the use of conventional synthetic pesticides and guarantee the safety of food and feedstuffs. However, the present study is only the first step towards the integration of FER in an eco-friendly preservative solution. In addition to toxicity studies, further experimental works are required to provide essential knowledge such as the effect of FER on OTA production by a wide range of producing fungal strains, the effect of environmental conditions on the “anti-ochratoxin” activity, the required formulation to potentiate the bioactivity of the hydroxycinnamic acid and protect it from degradation. Besides, before conversion of these first results into a commercial preservative solution, the efficacy of the FER-formulated treatment, demonstrated under laboratory conditions, should be assessed under real conditions in grain silos.

## 4. Materials and Methods

### 4.1. Materials

All phenolic acids were purchased from Sigma Aldrich (Saint-Quentin Fallavier, France). Solvents were purchased from VWR (Fontenay-Sous-Bois, France). Reagents were purchased from Scharlau (Barcelona, Spain), Sigma Aldrich (Saint-Quentin Fallavier, France) and Thermo Fischer Scientific (Illkirch, France). OTA standard was purchased from Romer Labs (Tulln, Austria).

### 4.2. Fungal Species

*A. westerdijkiae* (CBS 112803) and *P. verrucosum* (NRRL 5517) were used throughout the study. Stock cultures were maintained at 4 °C on potato dextrose agar (PDA) in Petri dishes. When inoculum was required, strain was grown on PDA slants at 25 °C in the dark for 5 days and spore suspensions were prepared by adding 6 mL of sterile distilled water to the PDA with gentle shaking.

### 4.3. Media and Culture Conditions

Five media prepared as described by Koteswara Rao et al. [23] with slight modifications were tested in this study:

1. AM medium: glucose 50 g, yeast extract 3 g, (NH_4_)_2_ SO_4_ 3 g, KH_2_PO_4_ 10 g, MgSO_4_.7H_2_O 2 g, distilled water 1000 mL.
2. CYA medium: sucrose 30 g, yeast extract 5 g, NaNO_3_ 0.5 g, KCl 0.05 g, MgSO_4_.7H_2_O 0.5 g, FeSO_4_.7H_2_O 0.01 g, K_2_HPO_4_ 1.3 g, CuSO_4_.5H_2_O 0.005 g, ZnSO_4_.7H_2_O 0.01 g, distilled water 1000 mL.
3. ME medium: sucrose 30 g, malt extract 40 g, peptone 1 g, CuSO_4_.5H_2_O 0.005 g, ZnSO_4_.7H_2_O 0.01 g, distilled water 1000 mL.
4. YES_A medium: sucrose 150 g, yeast extract 20 g, MgSO_4_.7H_2_O 0.5 g, CuSO_4_.5H_2_O 0.005 g, ZnSO_4_.7H_2_O 0.01 g, distilled water 1000 mL.
5. YES_B medium: sucrose 150 g, yeast extract 10 g, MgSO_4_.7H2O 2 g, CuSO_4_.5H_2_O 0.005 g, ZnSO_4_.7H_2_O 0.01 g, distilled water 1000 mL.

The five media were compared in sterile Petri dishes (55 mm diameter) for the static conditions and in 50 mL sterile conical centrifuge tubes for the agitated conditions. Static Petri dishes and conical tubes containing 8 and 20 mL medium, respectively, were inoculated with 2 × 10^4^ spores mL^−1^ and incubated in dark at 25 °C for 11 days. Conical tubes were incubated in a Multitron incubator (Infors AG, Bottmingen, Switzerland) with shaking at 150 rpm. Cultures implemented to compare media and static vs. agitated conditions were done in triplicate.

Studies with phenolic acids were conducted in static sterile Petri dishes (90 mm in diameter) containing 20 mL ME medium supplemented or not with 0.5 mM CHLO, CAF, FER, or COUM directly diluted in medium, for 11 days. For kinetic study, additional incubation times were included (2, 3, 4, 7, and 10 days). Appropriate non-inoculated controls with and without phenolic acids were performed. It was checked that the initial pH of the culture medium was not affected by the phenolic acid supplementation and that final pH conditions did not vary between treatments. Five replications were done for each condition.

Following incubation, cultures were centrifuged at 3000× *g* for 10 min. Supernatants were stored at −20°C before OTA analysis. Fungal biomass was measured by weighing the mycelial pellet after 48 h of freeze-drying. Dry mycelia were stored at −20 °C until analysis.

### 4.4. OTA Extraction and Measurement

In a 3 mL sample of culture supernatant, 20 µL of 37% HCl were added to adjust the pH value to 1–2. OTA was extracted with 4 mL of chloroform by agitating for 2 min. After centrifugation (10 min at 3000× *g*), 3 mL of the chloroform phase were evaporated to dryness under a nitrogen stream at 50 °C. The resulting residue was dissolved in 500 µL of methanol and filtered through a 0.2 µm filter before LC/FLD analysis.

OTA in mycelia was extracted by agitating 0.2–0.8× *g* of lyophilized ground mycelium with 20 mL of methanol during 60 min. After centrifugation (5 min at 3000× *g*), 5 mL were evaporated to dryness under a nitrogen stream at 50 °C. The resulting residue was dissolved in 500 µL of methanol and filtered through a 0.2 µm filter before liquid chromatography/fluorimetry detector (LC/FLD) analysis.

Quantification of OTA was performed on a Shimadzu Prominence Ultra Performance Liquid Chromatography (UPLC) chain, equipped with two pumps LC-30 AD, a degasser DGU-20A5R, an auto sampler SIL-30 AC, and a FLD detector RF-20A XS (Shimadzu Scientific Instruments, Noisiel, France). Separation was achieved on a Kinetex 2,6U column (150 × 4.6 mm; 2.6 µm) (Phenomenex, Le Pecq, France) maintained at 45 °C. The mobile phase consisted of 0.2% formic acid in water (*v/v*) (solvent A) and acetonitrile (solvent B). The following gradient was used for elution: 45% B for 3 min, 45–95% B in 1 min, 95% B for 1 min, 95–45% B in 0.5 min, and 3 min post-run equilibration with initial conditions. The flow was kept at 2 mL min^−1^. The injection volume was 0.5 μL. The fluorescence detector was set up at excitation and emission wavelength of λex = 332 nm and λem = 466 nm. Quantification was performed using external calibration with standard solutions.

### 4.5. Analysis of Phenolic Acids and Their Biotransformation Products

Phenolic acids and products resulting from FER and COUM biotransformation were directly analyzed by LC/DAD on an 1100 Series High-Performance Liquid Chromatography (HPLC) system (Agilent, Les Ulis, France) as described by Gauthier et al. [15].

### 4.6. Statistical Analyses

The significance of the effect of phenolic acids on fungal growth and OTA production by *A. westerdijkiae* and *P. verrucosum* were tested with Student’s *t* test (control vs. treated).

For kinetic study, one-way ANOVAs for OTA and fungal biomass were carried out. Differences between control and tested phenolic acids in terms of OTA were determined separately for each day with multiple comparisons tests using the Dunn–Sidak correction. Statistical analysis was performed using XLSTAT 2019 software (Addinsoft, Rennes, France). Level of significance was set at *p* = 0.05.

## Figures and Tables

**Table 1:** Fungal growth of *Aspergillus westerdijkiae* and *Penicillium verrucosum* cultivated in five different media under static and agitated conditions.

Media	Initial pH	*A. westerdijkiae*		*P. verrucosum*	
		Final pH	Dry Fungal Biomass ^1^, mg	Final pH	Dry Fungal Biomass ^1^, mg
**Static Condition**					
AM	5.1	2.0	202.37 ± 3.37	3.0	234.33 ± 29.40
CYA	7.2	8.0	170.33 ± 5.01	7.0	127.27 ± 1.72
ME	4.2	5.5	264.87 ± 3.89	5.0	235.93 ± 5.03
YES_A	6.6	6.0	506.50 ± 6.08	6.0	372.77 ± 7.06
YES_B	6.7	6.0	521.00 ± 53.66	4.5	426.67 ± 14.88
**Agitated Condition**					
AM	5.1	4.0	324.33 ± 33.01	4.5	432.37 ± 114.87
CYA	7.2	7.0	340.80 ± 25.64	6.0	383.63 ± 71.83
ME	4.2	4.0	609.70 ± 303.83	4.0	381.83 ± 57.87
YES_A	6.6	6.0	1484.33 ± 103.01	5.5	1135.37 ± 96.56
YES_B	6.7	4.0	1057.87 ± 43.37	5.5	880.77 ± 155.54

^1^ Data are means ± standard deviation using three biological replicates. AM, Adye and Mateles medium; CYA, Czapek Yeast Autolysate medium; ME, Malt Extract medium; YES_A, Yeast Extract Sucrose_A medium; YES_B, Yeast Extract Sucrose_B medium.

## Abbreviations

OTA	Ochratoxin A
Pv	*Penicillium verrucosum*
Aw	*Aspergillus westerdijkiae*
HBENZ	*p*-Hydroxybenzoic acid
CAF	Caffeic acid
CHLO	Chlorogenic acid
COUM	*p*-Coumaric acid
FER	Ferulic acid
VAN	Vanillic acid
LC/DAD	Liquid chromatography coupled with a Diode Array Detector
LC/FLD	Liquid chromatography coupled with a fluorescence detector
ANOVA	Analysis of variance
FAO	Food and Agriculture Organization
IARC	International Agency for Research on Cancer
EC	European Commission
AM medium	Adye and Mateles medium
CYA medium	Czapek Yeast Autolysate medium
ME medium	Malt Extract medium
YES_A medium	Yeast Extract Sucrose_A medium
YES_B medium	Yeast Extract Sucrose_B medium
